# Feasibility and preliminary efficacy of a physical activity counseling intervention using Fitbit in people with knee osteoarthritis: the TRACK-OA study protocol

**DOI:** 10.1186/s40814-015-0027-x

**Published:** 2015-08-22

**Authors:** Cam Clayton, Lynne Feehan, Charlie H. Goldsmith, William C. Miller, Navi Grewal, Joanna Ye, Ju Young Yoo, Linda C. Li

**Affiliations:** 1 Arthritis Research Canada, Milan Ilich Arthritis Research Centre, 5591 No. 3 Road, Richmond, BC V6X 2C7 Canada; 2 Department of Physical Therapy, University of British Columbia, Friedman Building, 212-2177 Wesbrook Mall, Vancouver, BC V6T 1Z3 Canada; 3 Rehabilitation Program, Fraser Health, Central City Office, 400-13450 102nd Avenue, Surrey, BC V3T 0H1 Canada; 4 Faculty of Health Sciences, Simon Fraser University, Blusson Hall, Room 9510, 8888 University Drive, Burnaby, BC V5A 1S6 Canada; 5 Department of Occupational Science & Occupational Therapy, University of British Columbia, T325-2211 Wesbrook Mall, Vancouver, BC V6T 2B5 Canada

**Keywords:** Knee osteoarthritis, Physical activity, Wearable activity tracker, Activity counseling, Feasibility

## Abstract

**Background:**

Physical activity (PA) reduces pain and improves functioning in people with knee osteoarthritis (OA), but few people with the condition meet recommended PA guidelines. Successful intervention strategies to increase PA include goal setting, action planning, self-monitoring, and follow-up feedback from a healthcare professional. Recently developed consumer wearable activity trackers allow users to set activity goals, self-monitor daily goal-progress, and provide feedback on goal attainment. It is hypothesized that a multi-component physiotherapist-led intervention that includes a short (40-min) education module, guided goal-setting and action planning, the use of a wristband activity tracker, and weekly follow-up phone calls will lead to increased PA outcomes.

**Methods/design:**

Thirty-six participants will be recruited from the community for a two-group pilot randomized controlled trial with a stepped-wedge design using an intention-to-treat analysis. Computer-generated block randomization will be performed using varying block sizes and a 1:1 allocation ratio. The 4-week intervention will be delivered immediately (immediate-intervention group) or after a 5-week delay (delayed-intervention group). Outcome measures of pain and disability (Knee Injury and OA Outcome Score), disease self-management ability (Partners in Health Scale), and objective bouted moderate-to-vigorous PA and sedentary time (BodyMedia SenseWear Mini Armband) will be collected at baseline (week 0) and two follow-ups (weeks 5 and 10), for a total study duration of 11 weeks. Feasibility data relating to process, resource, management, and scientific elements of the trial will be collected. Outcome measure and feasibility data will be summarized, and an estimate of intervention efficacy will be obtained by regression model with planned comparisons. The trial began recruiting in February 2015. To date, 34 subjects have been recruited.

**Discussion:**

This study will evaluate the feasibility and preliminary efficacy of a novel intervention to promote PA in people living with knee OA. The results will provide valuable information to inform a larger randomized trial to assess intervention effectiveness.

**Trial registration:**

ClinicalTrials.gov Identifier: NCT02313506 (registration date 8 December 2014). First participant randomized 20 February 2015.

## Background

Osteoarthritis (OA) is the most common chronic joint disease, affecting 10–15 % of people in North America [1]. The knee joint is most commonly affected, with knee OA a leading cause of chronic pain and functional limitation that can decrease one’s ability to participate in daily activities and reduce quality of life [2]. Due to increasing rates of obesity and aging population demographics, the prevalence of OA in Canada is projected to more than double within a generation with associated costs (CAD$27.5 billion in 2010) projected to increase to CAD$1.45 trillion by 2040 [3].

OA remains a condition with no cure, and current non-surgical treatments are aimed at alleviating symptoms while maintaining high quality of life. Physical activity (PA) is a core first-line treatment [4, 5], with both aerobic exercise and strength training known to safely reduce pain and improve functioning in this population [6–12]. A 2015 Cochrane review reported people with knee OA who exercised experienced a 12 % decrease in pain, 10 % improvement in physical function, and 4 % improvement in quality of life immediately following treatment [13]. Regular PA is also known to provide global health benefits that help manage or prevent chronic comorbidities such as hypertension, cardiovascular disease, or diabetes that commonly develop in people with this condition [14]. National PA guidelines state that all adults should participate in at least 150 min of moderate-to-vigorous PA (MVPA) per week in bouts of 10 min or more to obtain the health benefits of an active lifestyle [14].

Despite these recommendations, the knee OA population remains highly inactive. People living with knee OA spend roughly 10 h of their waking day in sedentary behaviours [15] and obtain 50 min per week of MVPA [16]. As few as 13 % of this population meet recommended activity guidelines [17, 18]. Insufficient PA in the knee OA population is particularly concerning given the emerging association of sedentary behaviour with poor health outcomes, including increased risk of metabolic syndrome, cardiovascular disease, diabetes, myocardial infarction, and all-cause mortality [19–22]. Reflective of this, Australian PA guidelines now recommend breaking up sitting time as often as possible throughout the day [23]. Greater levels of sedentary behaviour independent of MVPA participation have also been associated with poorer physical functioning in people with knee OA [15, 16]. As such, there is a clear need to promote greater activity levels and reduced sedentary time in this population.

PA promotion interventions among chronic disease populations have varied in format, setting, mode of delivery, and techniques employed to elicit behaviour change. A 2008 systematic review and meta-analysis found PA promotion interventions among chronically ill populations yielded a small-to-moderate effect size (ES = 0.45; 95 % confidence interval (CI) 0.38, 0.52) that was associated with 945 additional steps per day and 48 additional PA minutes per week. Interventions employing behaviour-oriented strategies such as goal setting, action plan formation, self-monitoring, and provision of feedback were associated with higher effect sizes than interventions focusing on education or cognitive strategies (such as motivational counseling or problem solving) alone [24]. Evidence has also been emerging supporting the use of wearable activity trackers such as pedometers or accelerometers for PA promotion. Activity trackers provide an objective measurement of activity output that facilitates goal setting and self-monitoring while serving as an environmental cue towards activity [25]. A 2009 meta-analysis of trials that used pedometers to promote PA found intervention participants took 2491 more steps per day relative to control groups (95 % CI 1098; 3885 steps per day) across eight trials, with significant but modest accompanying decreases in BMI and blood pressure [26]. Pedometer interventions have also been effectively employed in arthritis populations. For example, Talbot et al. combined a pedometer-driven walking program aimed at incrementally increasing daily step counts with self-management education for people with knee OA and compared this program to a group receiving self-management education alone. The intervention group achieved a 23 % increase in daily steps, as well as a statistically significant increase in isometric strength, suggesting arthritis populations may also benefit from the use of these devices [27].

Following recent technological improvements, sophisticated accelerometer-based activity trackers are now available for consumer use as motivational tools to increase PA. These devices offer a host of features unavailable to the pedometer, including the ability to track intensity of activities as opposed to steps only, and user-friendly smartphone or web-based interfaces that graphically depict activity over time. Modern activity trackers encourage users to set specific activity goals (e.g. number of steps) and monitor progress throughout the day, providing immediate feedback on goal achievement. As an additional benefit, web-based hosting and social network functionality of the user-interfaces facilitate ease of communication about activities between individuals and their healthcare team. Owing to their recent development, few studies report employing these devices as PA promotion tools; however, a systematic content analysis of consumer wearables concluded the devices featured a number of evidence-based techniques for promoting PA [25]. Additionally, results from studies in 2013 and 2015 employing a clip-on activity tracker noted significant increases in light, moderate, and vigorous PA and decreases in sedentary time [28, 29], whereas use of an armband activity monitor as a component of diet and PA interventions led to weight loss in overweight and obese subjects [30, 31]. While various models of wearable trackers are presently available (e.g. hip clip-ons, wristbands, ankle-straps), a popular consumer wristband accelerometer, the Fitbit Flex (Fitbit Inc., San Francisco, CA, USA), demonstrated high ratings of wearability and usability in a prior pilot study among arthritis patients conducted by our group [32]. Thus, the proposed study outlines a pilot randomized controlled trial of a novel PA intervention that leverages evidence-based behaviour-change techniques and the Fitbit Flex activity tracker to promote greater activity levels among people living with knee OA.

### Objectives and hypotheses

The primary objectives of the present study are to pilot and collect feasibility data on trial processes, resources, and management and to assess the preliminary efficacy of a PA promotion intervention for increasing bouted MVPA in people with knee OA. The proposed intervention consists of three components: (1) a 1.5-h in-person small group session including a physiotherapist-led education module, personalized activity counseling, and action planning; (2) the use of a Fitbit Flex activity tracker; and (3) weekly physiotherapist follow-up phone calls to modify activity levels as needed. Secondary objectives related to efficacy include assessing if the intervention decreases bouted sedentary time, improves knee OA disease severity, and improves perceptions of self-management ability.

It is hypothesized that the intervention will: (1) increase bouted MVPA time (bout ≥ 10 min), (2) decrease bouted sedentary time (bout ≥ 20 min), (3) increase perceptions of self-management ability, (4) decrease pain, and (5) increased functional ability in people with knee OA.

## Methods/design

This protocol is guided by the Standard Protocol Items: Recommendations for Intervention Trials (SPIRIT) 2013 guidelines [33]. This study has received approval from the University of British Columbia Clinical Research Ethics Board (H14-02631), and the study was registered on ClinicalTrials.gov (NCT02313506).

### Study design

**Fig. 1:**
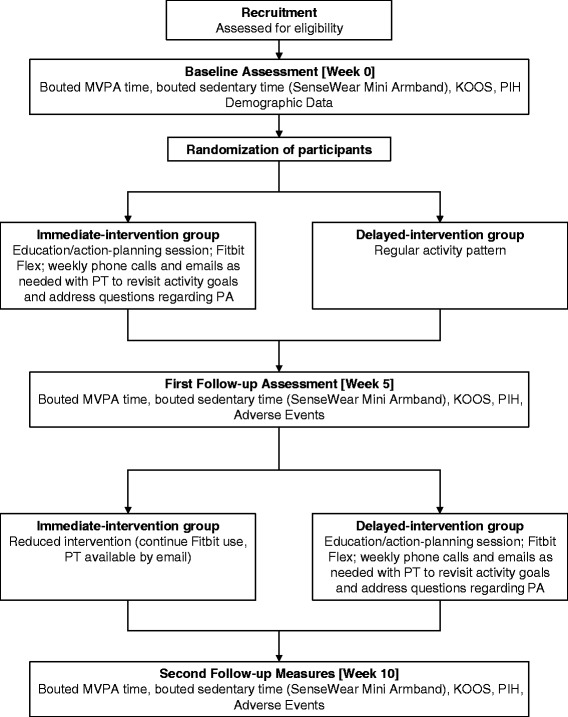
TRACK-OA study flowchart. Acronyms: *PA* physical activity, *PT* physiotherapist, *MVPA* moderate-to-vigorous physical activity, *KOOS* Knee Injury and Osteoarthritis Outcome Score, *PIH* Partners in Health Scale

The TRACK-OA study will be carried out at the Arthritis Research Canada (Richmond, BC, Canada) and the Mary Pack Arthritis Centre (Vancouver, BC, Canada). Participants will be recruited from the community in the Greater Vancouver Area. The study is a two-group pilot randomized controlled trial (RCT) with a stepped-wedge design. The stepped-wedge is a trial design in which all participants receive the intervention but the time point at which the intervention is received is randomized (immediate, or after a 5-week delay) (see Fig. 1 for trial flowchart). After receiving informed consent from the participants, baseline measures will be collected.

### Participants

A convenience sample of 36 participants will be recruited from the community by way of community posters and web-based advertisements. Interested participants will complete a web-based screening questionnaire and eligible participants are phoned for further screening, obtainment of informed consent, and study enrollment.

### Eligibility criteria

Participants are included if they (1) possess a physician-confirmed diagnosis of knee OA or are both over 50 years and have experienced 4 weeks of pain, aching, or discomfort in or around the knee during the last year (equal to or more than 28 separate or consecutive days) [34]; (2) have no previous diagnosis of inflammatory arthritis, connective tissue diseases, fibromyalgia, or gout; (3) have no history of using disease-modifying anti-rheumatic drugs or gout medications; (4) have no prior knee arthroplasty; (5) are not on the waitlist to receive total knee arthroplasty; (6) have no history of acute knee injury in the past 6 months; (7) have not had lower extremity or back surgery in the past 12 months; and (8) have an email address and daily access to a personal computer with internet access.

Individuals are excluded if they (1) have a BMI of ≥40 kg/m^2^, (2) have received a steroid injection in a knee in the last 6 months, (3) have received a hyaluronate injection in a knee in the last 6 months, (4) are using medications that impair activity tolerance (such as beta-blockers), and (5) have an inappropriate level of risk for increasing their unsupervised PA as identified by the Physical Activity Readiness Questionnaire (PAR-Q) 2014 [35]. Specifically, if an individual answers “Yes” to any conditions described in the General Health Questions section, they will complete a series of standardized follow-up questions. Any individual who does not pass the PAR-Q will require physician clearance to participate, and those with severe health issues such as a heart or cardiovascular condition will be deemed ineligible under this criterion. In some cases, an individual may fail the PAR-Q on the basis of their knee OA alone. In these instances, further clarification questions will be asked to determine whether physician clearance is required as per the procedure outlined in Table 1.

**Table 1:** An example of applying the PAR-Q to determine eligibility for the randomized controlled trial

PAR-Q questions	Action
General health section	
6. Do you currently have (or have had within the past 12 months) a bone, joint, or soft tissue problem that could be made worse by becoming more physically active?	If “yes” due ONLY to knee OA, answer follow-up questions 1a and 1b of PAR-Q
	If “yes” due to other problems, complete entire follow-up section
Follow-up section	
1a. Do you have difficulty controlling your condition with medications or other physician-prescribed therapies?	If “yes”, the person will require physician clearance
	If “no”, the person passes this criterion
1b. Do you have joint problems causing pain, recent fracture or fracture caused by osteoporosis or cancer, displaced vertebra, and/or spondylolysis/pars defect?	If “yes” ONLY to knee OA causing pain, then ask clarifying questions below
	If “yes” to other conditions, the person will require physician clearance
Clarifying questions	
1. How severe are your symptoms after physical activity?	If mild symptoms for <2 h, no physician clearance necessary
2. After physical activity, do your symptoms last longer than 2 h?	Otherwise, require physician clearance

### Outcome measures

#### Descriptive measures

Demographic information including age, gender, income, level of education, height, weight, presence of comorbidities, whether an individual received a physician diagnosis of knee OA, and time since onset are collected to describe the sample and compare group randomization at baseline.

#### Feasibility data

To address the primary objective, data relating to trial feasibility will be collected. As suggested by Thabane et al. [36], these data will be been organized according to process, resources, management, and scientific issues. Setting benchmarks for feasibility data is beneficial to inform larger-scale assessments of this intervention in the future [36]. Thus, benchmark criteria for success were set in accordance with the goals of an upcoming proof-of-concept study featuring the present intervention (see Table 2). Detailed information on the recruitment process will be collected, including the number of individuals who contacted us, the source of recruitment, and the reason for non-eligibility or withdrawal of interest. Adherence to study protocols (education and training session attendance, telephone follow-up, Fitbit use, outcome measure collection) will be assessed for each participant by determining whether a participant completed each component of the study. It has been determined that at least 4 days of data are required to obtain accurate estimates of PA participation from the SenseWear armband [37]. Time to complete online questionnaires will be collected from the online survey system. As the cost of activity monitors (Fitbit and SenseWear) in this study is a concern, extremely high equipment retention and reliability is required. Management issues such as unforeseen challenges with the host location, study personnel, or data collection that challenge study logistics or success will be carefully documented. Safety issues and adverse events will be tracked throughout the study (see “Risk management” subsection below). Feasibility data will be tracked and logged in a detailed administrative database and summarized for presentation at study completion. Treatment effect and variance estimates will be calculated in order to inform sample size calculations for the larger trial. These calculations will be carried out assuming a randomized trial with two equal-sized groups, a two tailed significance threshold (alpha) of 0.05, and power of 0.8 to detect a significant result between groups on bouted MVPA and sedentary time outcomes.

**Table 2:** Feasibility data to be collected and criteria for success

Feasibility data category	Item of interest	Success criteria
Process	Recruitment rate	2 participants/week
	Consent rate	≥90 %
	Dropout rate	≤10 %
	Adherence to protocols	4 days × 20 h for each SenseWear week; 100 % completion of online questionnaires; mean 5 days/week of Fitbit use
Resources	Equipment loss/reliability	100 % equipment retention
	Personnel	Sufficient personnel to execute the trial efficiently
Management	Location, logistics, personnel, data	No criteria set
Scientific	Intervention effect, variation, safety	No criteria set

### Patient-centered outcome measures

#### Primary outcome

##### Bouted MVPA time

Bouted MVPA time will be measured by the BodyMedia SenseWear® Mini armband (Pittsburgh, PA, USA). The SenseWear armband uses multi-modal information and proprietary algorithms to estimate 24-h free-living activity levels (steps, energy expenditure, vigorousness of activity). Measurements are recorded in 1-min epochs from the device’s triaxial accelerometer and sensors detecting galvanic skin response, skin temperature, and heat flux. It is initialized using the proprietary SenseWear software, and the armband is placed comfortably on the upper triceps and worn all hours of the day except during water-based activities. The SenseWear assesses PA vigorousness in terms of metabolic equivalent of task (MET). Activities ≥3.0 METs are considered MVPA, thus MVPA is operationalized as any activity that generates an output of ≥3.0 METs for bouts of 10 min or more as assessed by the SenseWear armband. Bouts of MVPA lasting ≥10 min are selected as the outcome measure of interest (as opposed to total MVPA time) because of the health promoting benefits of this activity pattern as recommended by national PA guidelines [14]. Allowing for up to a 2-min drop below MVPA threshold has been proposed as a reasonable approach for coping with real-world scenarios that may interrupt PA (i.e. waiting to cross the road at a stop light for 1 min while walking) [38] and has been employed in the national PA assessment studies using accelerometers [39, 40]. Thus, dropping below 3.0 METs for more than 2 min within a bout of activity lasting 10 min or more will constitute the end of a bout of MVPA [41].

#### Secondary outcomes

##### Bouted sedentary time

Sedentary behaviour is defined as any waking behaviour characterized by an energy expenditure ≤1.5 METs while sitting or reclining [42]. Because prolonged sedentary behaviour is of primary concern [23], sedentary time is operationalized as any wakeful (e.g. non-sleeping) behaviour generating ≤1.5 METs for a continuous bout of ≥20 min as assessed by the SenseWear armband.

##### Disease status

Measures of knee OA disease status are assessed with the Knee Injury and OA Outcome Score (KOOS). The KOOS questionnaire was developed for use in a population recovering from knee injury but has been shown to be a reliable and valid measure of knee status for the OA population [43–45]. The questionnaire makes use of five separately interpreted subscales that assess (1) pain, (2) other symptoms, (3) function in daily life, (4) function in sport and recreation, and (5) knee-related quality-of-life. Each of these subscales is assessed by a five-point Likert scale from 0 to 4, with 0 being “No Problems” and 4 being “Extreme Problems”. Each subscale score is transformed to a 0–100 scale, with 0 representing no knee problems and 100 representing extreme knee problems. The KOOS contains all items from the Western Ontario McMaster OA Index [46] in its original form but also includes questions about functioning in sports and recreation as well as quality of life, both particularly relevant for the purposes of this intervention [47].

##### Disease self-management

The Partners in Health Scale (PIH) is used to assess changes in participant perceptions of self-management ability. The PIH was developed to provide a generic tool for objective assessment of patient self-management skill to provide more targeted self-management interventions to those who most need it. The self-rated scale includes 12 items that probe six principles of successful chronic disease self-management, including whether patients (1) have knowledge of their condition; (2) follow a treatment plan agreed on with healthcare professionals; (3) actively share in decision-making with healthcare professionals; (4) monitor and manage signs and symptoms of their condition; (5) manage the impact of conditions on the physical, emotional, and social aspects of life; and (6) adopt lifestyles that promote health. Each item is rated on an eight-point scale, with 0 indicating the highest self-management and 8 indicating the lowest self-management (min-max 0–96). The PIH has been shown to have good internal consistency (Cronbach’s α = 0.82) and construct validity and is a suitable outcome measure for measuring change in patient self-management knowledge and behaviour over time [48].

#### Assessments

Assessment of outcome measures will occur at three time points—baseline (T0), week 5 (T1), and week 10 (T2)—with the exception of demographic information (collected at baseline only) and adverse events (collected at T1 and T2 only). Both the KOOS and PIH will be uploaded to an online survey system hosted on a secure local server at Arthritis Research Canada, and the SenseWear armbands will be delivered to participants prior to each data collection week.

#### Randomization

**Fig. 2:**
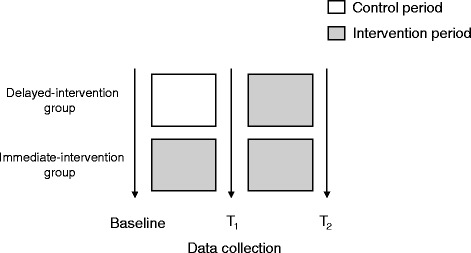
TRACK-OA randomization diagram. T_1_ data collection occurs during week 5; T_2_ data collection during week 10

Once informed consent is received and baseline measures have been collected, participants will be randomly assigned to one of two groups (immediate-intervention group or delayed intervention group). As per the stepped-wedge design, the delayed-intervention group will receive the intervention after a 5-week waiting period (i.e. immediately following T1 follow-up assessment; see Fig. 2). A study statistician (CHG) not involved in the day-to-day operation of the trial will randomly assign participants in blocks to each group using a computer-based random number generator and a 1:1 allocation ratio. Block sizes will be concealed from study staff and randomly varied to prevent prediction of group allocation. The statistician will pass group allocations back to study research coordinators (CC, NG) who will arrange for intervention delivery as appropriate. Group assignments will be accessible only to the study research coordinators.

#### Intervention

##### Education and training session

The education and training session are delivered at the Mary Pack Arthritis Center by a physiotherapist and research staff member to groups of two to four participants. The education module runs for roughly 40 min. A study physiotherapist leads a discussion on the causes and nature of knee OA, cornerstone treatments of the disease, and the role of PA and exercise in the management of OA. Common myths about knee OA are discussed and dispelled (e.g. “exercise makes my knee OA worse”), and a discussion of various types of PA and exercise appropriate for individuals with the condition are given. In addition, the emerging risks of a sedentary lifestyle are presented, and the importance of reducing sedentary time is emphasized. Finally, symptom-management strategies are discussed, including how to manage common problems that may arise when becoming active. Increasing activity by “listening to one’s body” is a key message in the education module, and ultimately the intent is to increase the participant’s knowledge about how to be physically active while having knee OA. Following the education module, the Fitbit Flex is given to each participant and 30-min training is provided. Participants then work individually with the physiotherapist to set personalized PA goals and create a weekly action plan. This component of the intervention is derived from Brief Action Planning—a structured, patient-centered method of setting goals and developing action plans based on the principles of motivational interviewing. Brief Action Planning first evokes a participant’s ideas of goals that are personally suitable, builds a specific action plan, and finishes by evoking a confidence statement (0–10 scale) [49]. A “barrier identification/problem solving” step was also added to the Brief Action Planning framework, which the physiotherapist will use to direct participants to set goals related to increasing MVPA participation and interrupting sedentary time. The education and training session will take an estimated 1.5 h.

##### Fitbit flex

Following the education and training session, participants will be asked to wear the Fitbit 24 h/day (unless charging) for the remainder of the study. The Fitbit Flex is a removable tracker (housing a triaxial accelerometer and five-light LED display) inside of a wristband worn on the non-dominant wrist. The Fitbit user-interface is an easy-to-use web-based application that graphically displays a user’s activities over the course of the day including total steps taken, calories burned, and distance traveled. Interaction with the user-interface may occur on a self-directed basis.

##### Telephone follow-up

Participants will be asked to share their Fitbit activity data with the physiotherapist they work with during the education session. This is accomplished using Fitbit’s online social networking function where users can privately share Fitbit activity “profile” with Fitbit “Friends”. Participants add the physiotherapist as a “Friend”, thus allowing the physiotherapist to view the participant’s activity data. These data will be used to inform the content of a 20-min weekly phone call to each participant from the physiotherapist (for the first 4 weeks of the intervention only). Like the goal setting portion of the education session, these conversations are derived from a Brief Action Planning protocol and are designed to respect participant autonomy while increasing confidence in PA participation. Furthermore, the physiotherapist may be contacted by email to address participant questions throughout the duration of the intervention as they arise.

#### Risk management and safety monitoring

Adverse events during the study will be tracked both by self-report on the online outcome measure questionnaire and by study physiotherapists during follow-up phone calls. In the online questionnaire, participants are asked if they experienced any negative outcomes (presented as a list of outcomes and allowing for free form entry at the end of the questionnaire) as a result of becoming more physically active since their enrollment in the study. They are asked to specify when the event occurred and if the event was experienced before. In an open-format response, participants are asked to describe the symptoms they experienced, any medical help they sought as a result, and any other relevant information. Any participants reporting adverse events will be immediately contacted and, if necessary, assessed by a study physiotherapist before being referred for appropriate care as needed.

#### Data management

Data will be stored in confidential servers on the Arthritis Research Canada premises. Participant ID’s are assigned upon enrollment, with a secondary ID assigned upon randomization. To ensure anonymity, participants are referred to by their study ID in emails and verbal communications where necessary. Access to all computer files with participant information will be limited to the study team, and individual files will be password protected. SenseWear data will be downloaded by a staff member (JY), who is blinded to participant’s group assignment, using SenseWear Professional Software 8.0, and exported to Excel (Microsoft, Redmond, WA) and processed in MatLab (The MathWorks, Inc., Natick, MA, USA). Data quality will be monitored on an ongoing basis by study staff, and backups will be saved weekly. The final de-identified data set will be provided to a blinded study statistician for analysis.

#### Sample size calculation

As pilot studies are primarily concerned with feasibility and generating estimates that will inform future studies’ power and sample size calculations, sample size, or power calculations were not deemed necessary for this trial [47]. A convenience sample of 36 participants was estimated to allow sufficient piloting of study protocols and estimates of feasibility measures to inform a future trial.

#### Statistical analysis

Descriptive measures will be tabulated and summarized by count, percentage, or mean, and 95 % CI and compared for differences between groups. Integrity of the randomization process will be monitored. SenseWear armband measurements for each time point will be considered valid if they include at least 4 days with at least 20 h of wear time each day [50]. Feasibility data will be collated and compared against benchmarks for success. When assessing preliminary intervention efficacy, the stepped-wedge design allows for analyses of differences between groups as well as temporal patterns. Changes in bouted MVPA, bouted sedentary time, KOOS score, and PIH score will assessed by a multiple regression model including variables for group, time, and group-time interaction. The effect of blocking will be considered in the analysis. Planned comparisons will be used to compare group means for differences at first follow-up (week 5) and to test for a delay effect and for temporal changes intervention efficacy. All analyses will be conducted using IBM SPSS Version 20 (Armonk, NY, USA).

#### Dissemination and knowledge translation

Patient collaborators from the Arthritis Research Canada’s Arthritis Patient Advisory Board (APAB) were consulted during the study design process to provide insight into patient values and concerns regarding physical activity and use of wearable activity trackers. Open channels of communication will be maintained with APAB during the study for patient perspectives on challenges or changes to study protocol. Study results will be featured on the Arthritis Research Canada website, as well as the lab website hosted through the University of British Columbia. Furthermore, results will be disseminated through the Arthritis Research Canada monthly e-newsletter and through a presentation at an APAB monthly meeting. Results will be shared with the scientific community by way of publication and conference presentation.

## Discussion

This paper presents a pilot RCT protocol to assess the feasibility of an intervention designed to increase MVPA and decrease sedentary time in a sample from the knee OA population. The program guides participants in setting personally relevant activity goals based on their preferences and level of ability and slowly increase PA participation over time. It is hoped that this patient-centered approach of gradually integrating greater activity into one’s existing life framework may enhance adherence to PA over time, a recognized challenge [6]. A potential limitation of this intervention is the technological skill requirement, which could act as a barrier to some individuals. However, there are several strengths. First, the use of the stepped-wedge trial design preserves the integrity of a randomized experiment while not withholding a potentially beneficial intervention from study participants. Conventional cross-over designs are impractical for PA promotion interventions as washout-period lengths have not been established and may be of considerable duration. Furthermore, the stepped-wedge design allows for assessment of lag-time effects, which may be relevant for people with arthritis as they may experience delays in the delivery of care. Piloting this trial format will be important in determining any feasibility challenges (e.g. delayed-group participant dropout) that may need addressing in future studies. Second, there is evidence to suggest the emphasis on action-oriented behavioural strategies for PA promotion may be more effective than cognitive or educational methods [24]. Third, the enhanced ability for participants to self-monitor goal progress and receive ongoing feedback that comes through the use of an activity tracker may increase awareness of PA behaviour and enhance activity-related self-efficacy, a known determinant of PA [51]. Fourth, the sharing of Fitbit data between participants and study physiotherapists encourages accountability and provides an opportunity to deliver more specific and personally relevant follow-up support to participants throughout the study. The feasibility metrics such as recruitment rate, dropout rate, adherence, and resource requirements collected in this study will be used to estimate the time, resources, and sample size required for a full-scale RCT. Furthermore, the experience gained from executing study protocols and the feedback provided from staff and participants will allow for improvement and refinement of trial elements and procedures to ensure the success of a future study to test the efficacy of this intervention. Pending the results of this pilot study, a full-sized trial will be designed and conducted in the Greater Vancouver Area in late 2015 and early 2016.

## Trial status

The trial began recruitment in 20 February 2015 and is presently open for recruitment. Recruitment will close when 36 participants have been randomized. It is anticipated this target will be met in August 2015.

## Abbreviations

BMI: body mass index
CI: confidence interval
KOOS: Knee Injury and Osteoarthritis Outcome Score
MET: metabolic equivalent
MVPA: moderate-to-vigorous physical activity
OA: osteoarthritis
PA: physical activity
PIH: partners in health scale
